# Prevalence of Mpox Vaccine Acceptance Among Students: A Systematic Review and Meta-Analysis

**DOI:** 10.3390/vaccines13020183

**Published:** 2025-02-13

**Authors:** Ambanna Yappalparvi, Shilpa Gaidhane, G. Padmapriya, Irwanjot Kaur, Madan Lal, Suhaib Iqbal, G. V. Siva Prasad, Atreyi Pramanik, Promila Sharma, Praveen Malik, Teena Vishwakarma, Ankit Punia, Megha Jagga, Rachana Mehta, Sanjit Sah, Muhammed Shabil, Prakasini Satapathy, Ganesh Bushi, Ali Davod Parsa, Russell Kabir

**Affiliations:** 1 Noida Institute of Engineering and Technology, Greater Noida 201306, India; ambanna910@gmail.com; 2 One Health Centre, Jawaharlal Nehru Medical College, Datta Meghe Institute of Higher Education, Wardha 442004, India; shilpa.gaidhane@dmiher.edu.in; 3 Department of Chemistry and Biochemistry, School of Sciences, JAIN (Deemed to be University), Bangalore 560069, India; g.padmapriya@jainuniversity.ac.in; 4 Department of Allied Healthcare and Sciences, Vivekananda Global University, Jaipur 303012, India; irwanjotkaur850@gmail.com; 5 Department of Medicine, NIMS University, Jaipur 303121, India; madan.lal1@nimsuniversity.org; 6 Chandigarh Pharmacy College, Chandigarh Group of College, Jhanjeri, Mohali 140307, India; suhaib2956.research@cgcjhanjeri.in; 7 Department of Chemistry, Raghu Engineering College, Visakhapatnam 531162, India; sivaprasad.gv@raghuenggcollege.in; 8 Division of Research and Innovation, School of Applied and Life Sciences, Uttaranchal University, Dehradun 2480072, India; atreyipram91@gmail.com; 9 Department of Microbiology, Graphic Era (Deemed to be University), Clement Town, Dehradun 248002, India; promilasharma.bt@geu.ac.in; 10 New Delhi Institute of Management, Delhi 110062, India; praveen.malik@ndimdelhi.org; 11 IES Institute of Pharmacy, IES University, Bhopal 462044, India; teena.research@iesuniversity.ac.in; 12 Centre of Research Impact and Outcome, Chitkara University, Rajpura 140417, India; ankit.punia.orp@chitkara.edu.in; 13 Chitkara Centre for Research and Development, Chitkara University, Rajpura 174103, India; megha.jagga.orp@chitkara.edu.in; 14 Clinical Microbiology, RDC, Manav Rachna International Institute of Research and Studies, Faridabad 121004, India; mehtarachana89@gmail.com; 15 Dr Lal PathLabs-Nepal, Chandol-4, Maharajgunj, Kathmandu 44600, Nepal; 16 SR Sanjeevani Hospital, Kalyanpur 56517, Nepal; sanjitsahnepal561@gmail.com; 17 Department of Paediatrics, Dr. D. Y. Patil Medical College, Hospital and Research Centre, Dr. D. Y. Patil Vidyapeeth, Pune 411018, India; 18 Department of Public Health Dentistry, Dr. D.Y. Patil Dental College and Hospital, Dr. D.Y. Patil Vidyapeeth, Pune 411018, India; 19 Center for Global Health Research, Saveetha Medical College and Hospital, Saveetha Institute of Medical and Technical Sciences, Saveetha University, Chennai 600077, India; mohdshabil99@gmail.com; 20 University Center for Research and Development, Chandigarh University, Mohali 140413, India; prakasini.satapathy@gmail.com; 21 Medical Laboratories Techniques Department, AL-Mustaqbal University, Hillah 51001, Iraq; 22 School of Pharmaceutical Sciences, Lovely Professional University, Phagwara 144411, India; ganeshbushi313@gmail.com; 23 School of Allied Health, Anglia Ruskin University, Essex, Chelmsford CM1 1SQ, UK; ali.parsa@aru.ac.uk

**Keywords:** Mpox vaccine, acceptance, hesitancy, systematic review, meta-analysis

## Abstract

Background: Mpox, formerly known as monkeypox, is a re-emerging viral disease. Vaccine acceptance is crucial for preventing its spread. This systematic review and meta-analysis assessed the acceptance of the Mpox vaccine among student populations. Methods: We searched electronic databases, including PubMed, Web of Science, and Embase, up to 14 September 2024. The studies included were observational, such as cross-sectional and cohort studies, and specifically assessed vaccine acceptance for Mpox vaccines among students. R version 4.4 was used to perform the meta-analysis, and sensitivity analyses were conducted to assess the robustness of the findings. The publication bias was evaluated using Doi plots. Results: Of the 143 studies initially identified, eight studies were included in the final analysis, comprising a total of 16,129 participants. The overall vaccine acceptance rate was 58.6%, with considerable variability across studies (I^2^ = 100%). The sensitivity analyses indicated that acceptance rates ranged between 45% and 70%. The Doi plot demonstrated the presence of moderate publication bias. Conclusion: This systematic review and meta-analysis shows moderate acceptance of the Mpox vaccine among students. Future studies should investigate the factors influencing vaccine acceptance and design targeted strategies to improve coverage, which will be essential for controlling Mpox and ensuring successful vaccination campaigns.

## 1. Introduction

Mpox, previously known as monkeypox, has re-emerged as a major public health issue, affecting regions both where the disease is endemic and where it is not commonly found [1]. First discovered in 1958, Mpox has recently resurfaced, leading global health organizations like the World Health Organization (WHO) to stress the need for vaccination as a primary way to prevent its spread [2]. While having vaccines available is crucial, the overall success of vaccination campaigns largely depends on how willing the public is to receive the vaccine [3]. University and college students are a particularly important group for disease control because they live in close proximity to one another and engage in frequent social activities, which can increase the risk of spreading diseases like Mpox [4].

Several factors influence vaccine acceptance, including perceived risk, trust in healthcare systems, the availability of reliable information, and cultural or social attitudes [5,6,7]. The recent COVID-19 pandemic highlighted the challenges of vaccine hesitancy, showing how misinformation and a lack of trust can interfere with public health initiatives [8,9]. The research conducted during the pandemic identified key factors that affect vaccine acceptance, such as individuals’ sense of vulnerability to the disease, confidence in vaccine safety, and influence from peers [10]. However, much of this research focused on COVID-19, and there is a limited understanding of how these factors apply to Mpox vaccine acceptance, especially in student populations [11,12]. Mpox presents distinct challenges due to its zoonotic origins and less widespread public awareness, which may alter how students perceive the disease and their willingness to get vaccinated [13].

Given the unique characteristics of Mpox and the important role students play in preventing its spread, a thorough review of Mpox vaccine acceptance among this population is needed. This systematic review and meta-analysis aims to fill this gap by assessing the existing data on student attitudes toward Mpox vaccination. The findings will provide valuable insights to inform and guide targeted public health strategies aimed at increasing vaccine uptake among students.

## 2. Methods

This systematic review and meta-analysis was conducted in accordance with the PRISMA guidelines (https://www.prisma-statement.org/prisma-2020, (accessed on 9 December 2024)) (Table S1) [14]. The protocol was registered with PROSPERO (https://www.crd.york.ac.uk/prospero/display_record.php?RecordID=586344, (accessed on 9 December 2024)) (CRD42024586344).

### 2.1. Eligibility Criteria

Studies were included if they employed observational designs, such as cross-sectional or cohort studies, and provided quantitative data on Mpox vaccine acceptance among students in post-secondary or higher education. Studies focusing on general vaccine attitudes without specific data on Mpox acceptance, letters to the editor, commentaries, qualitative studies, abstract-only publications, case series, case reports, reviews, and discussion papers were excluded (Table S2).

### 2.2. Search Strategy

We performed comprehensive searches across electronic databases, including PubMed, Web of Science, and Embase, up to September 2024, to identify relevant studies. The search strategy utilized a combination of keywords and Boolean operators. Specifically, the search terms used were “Mpox” OR “Monkeypox” AND “acceptance” OR “uptake” AND “students” OR “medical students” (Table S3).

### 2.3. Screening and Data Extraction

The screening process involved two phases using Nested Knowledge software (https://nested-knowledge.com/gather, accessed on 9 December 2024). Two independent reviewers initially screened the titles and abstracts for relevant studies based on predefined eligibility criteria. The selected studies were then retrieved for full-text screening based on predefined eligibility criteria. Disagreements were resolved by a third reviewer at both stages. 

The data extraction was performed using Nested Knowledge software to ensure a structured and efficient process. Two authors independently extracted data from each study that met the inclusion criteria. The information collected included specific study characteristics such as the country, the study design, the sample size, demographics of the participants, and vaccine acceptance rate. The discrepancies in data extraction between the two authors were resolved through discussion or, if necessary, consultation with a third author to reach a consensus.

### 2.4. Quality Assessment

The Modified Newcastle-Ottawa Scale (NOS) was used to assess the quality of observational studies, evaluating the representativeness, sample size (<500), Mpox definition, and outcome ascertainment. High-quality studies received 5–6 stars, indicating strong methodology, while moderate-quality studies scored 4–5 stars, reflecting some limitations. Low-quality studies, with 0–3 stars, displayed significant flaws, such as poor participant selection or inadequate follow-up (Table S4) [15].

### 2.5. Statistical Analysis

The meta-analysis was conducted using R version 4.4 software [16]. The I^2^ statistic was employed to assess heterogeneity across studies, with low heterogeneity (0–40%) leading to the use of a fixed-effect model and high heterogeneity (75–100%) requiring a random-effects model [17]. Leave-one-out sensitivity analyses were conducted by excluding individual studies one at a time to evaluate the robustness of the results [18]. Publication bias was assessed through Doi plots and the Luis Furuya-Kanamori (LFK) index [19].

## 3. Results

**Figure 1:**
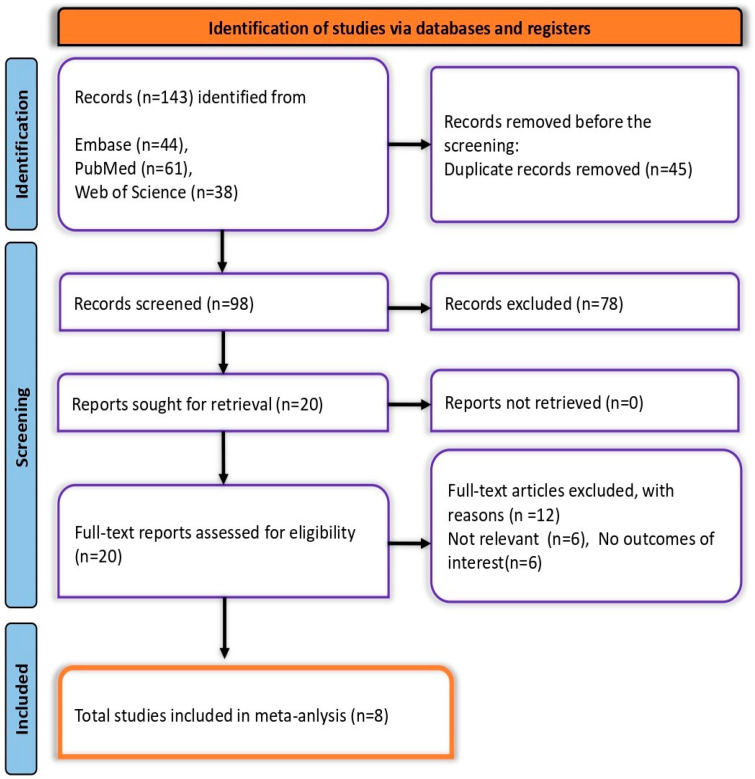
PRISMA flow diagram depicting the screening and selection process.

A total of 143 records were identified: 61 from PubMed, 44 from Embase, and 38 from Web of Science, along with two additional records from citation searching. After removing 45 duplicates, 98 records remained for screening. Of these, 78 were excluded during the initial screening, leaving 20 reports for full-text assessment. Twelve were further excluded due to irrelevance. Ultimately, eight studies met all eligibility criteria and were included in the final meta-analysis (Figure 1).

### 3.1. Summary Characteristics of Included Studies

Eight cross-sectional studies on Mpox vaccine acceptance among students included 16,129 participants, of whom 8319 were male. The sample sizes varied significantly, ranging from 196 to 4380 participants. The ages of the participants ranged from under 16 to over 30 years. These studies were conducted across several nations, including Saudi Arabia [20], Egypt [21], USA [22], Pakistan [23], Algeria [24], and Germany [25], along with two studies from China [13,26] (Table 1). The Modified NOS revealed that the quality of the studies was moderate to high.

### 3.2. Meta-Analysis

**Figure 2:**
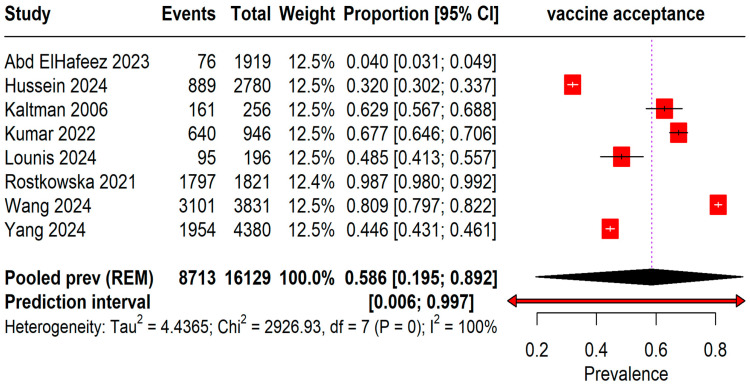
Forest plot illustrating Mpox Vaccine Acceptance among students [13,20,21,22,23,24,25,26].

The pooled prevalence of acceptance rates among 16,129 students was found to be 58.6% (95% CI: 19–89%) with an I^2^ = 100%. A wide range of prediction intervals was observed from 0.6% to 99.7%, indicating substantial variability in acceptance rates across different studies (Figure 2).

### 3.3. Subgroup Analysis

The pooled prevalence of vaccine acceptance is based on the geographic region and quality of the studies, using the NOS for quality assessment. Geographic variations were significant (Table 2). Saudi Arabia reported the lowest vaccine acceptance at 3.96% (95% CI, 3.13% to 0.29%), while Germany exhibited the highest at 98.68% (95% CI, 98.05% to 99.15%). The United States and Pakistan also showed high acceptance rates of 62.89% (95% CI, 56.66% to 68.82%) and 67.65% (95% CI, 64.57% to 70.63%), respectively. Subgroup analysis by study quality revealed that high-quality studies, comprising two studies with a total sample of 4601, had a notably higher pooled prevalence of 85.53% (95% CI, 0.00% to 100%), compared to moderate-quality studies, which included six studies with 11,528 participants and showed a pooled prevalence of 46.90% (95% CI, 13.96% to 82.78%). These results suggest a potential influence of both geographic and methodological factors on reported vaccine acceptance rates, indicating that higher-quality studies tend to report greater vaccine acceptance.

### 3.4. Sensitivity Analysis

**Figure 3:**
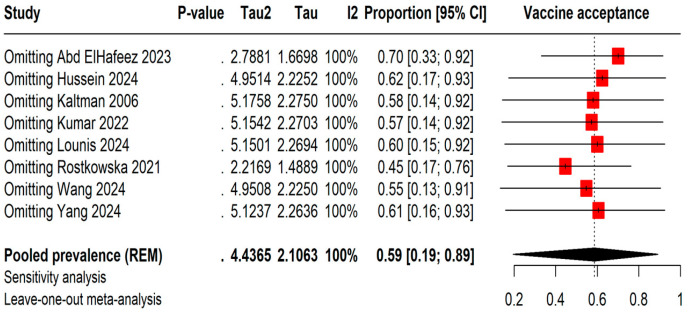
Leave-one-out sensitivity analysis of Mpox vaccine acceptance among students [13,20,21,22,23,24,25,26].

The leave-one-out meta-analysis shows that removing different studies causes the vaccine acceptance rates to vary between 45% by omitting Rostkowska et al., 2021 [25] and 70% by omitting Abd ElHafeez et al., 2023 [20]. However, even with these changes, the I^2^ statistic stays at 100%, indicating consistently high heterogeneity among the studies (Figure 3).

### 3.5. Publication Bias

**Figure 4:**
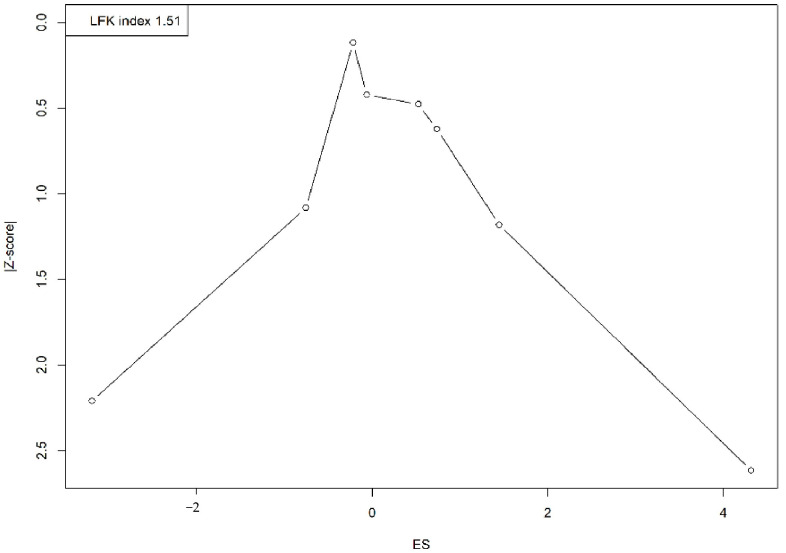
Doi plot representing the publication bias.

A visual assessment of the Doi plot revealed the presence of publication bias, confirmed by an LFK index of 1.51. The asymmetrical distribution, with studies clustering on one side, suggests an overrepresentation of smaller studies reporting larger or more significant effects, indicating potential publication bias or small-study effects (Figure 4).

## 4. Discussion

This systematic review and meta-analysis provides a comprehensive assessment of Mpox vaccine acceptance among student populations, revealing a moderate acceptance rate of 58.6%. The wide prediction interval (0.006 to 0.997) and high heterogeneity (I^2^ = 100%) highlight the variability in acceptance across different studies, as well as the diverse factors that influence vaccine uptake among students. These factors may include cultural differences, access to healthcare, perceptions of disease risk, and trust in public health authorities. Given the resurgence of Mpox and the critical role students play in controlling infectious disease spread, understanding these determinants is crucial for improving vaccine acceptance.

A prior study on COVID-19 vaccine acceptance was examined alongside the meta-analysis results. Roy et al., 2023 reported high vaccine acceptance rates among university students, with 88.1% acceptance among public university students [27]. Key influences included trust, communication, safety, efficacy, and political roles. Similarly, Patwary et al., 2022 conducted a systematic review of healthcare students, reporting a global vaccine acceptance rate of 68.8%, with substantial country-specific differences [28]. Both this study and the current meta-analysis observed variability in vaccine acceptance among students. In another study, Geng et al. (2023) examined vaccine acceptance among college students, identifying trust, communication, safety, and government policies as key factors influencing acceptance [29]. The study found that urban males were more likely to accept the vaccine compared to rural females, emphasizing the role of sociodemographic factors in vaccine uptake. While both Geng’s study and the meta-analysis identified external factors as important determinants, Geng’s study focused on specific demographics, whereas the meta-analysis provided a global overview of vaccine acceptance variability. Kelekar et al. (2021) studied vaccine acceptance among U.S. dental and medical students, finding that dental students (45%) were more hesitant to receive the COVID-19 vaccine compared to medical students (23%) [30]. This rate was lower than the 58.6% reported in the meta-analysis. Professional exposure and education influenced acceptance, with medical students more likely to support mandatory vaccination and trust public health information.

The studies on vaccine acceptance show distinct differences. Kanyike et al. (2021) reported a 37.3% acceptance rate among Ugandan medical students, influenced by factors like gender, relationship status, and social media [31]. In contrast, Sulaiman et al. (2024) found a higher 67% acceptance rate among people living with HIV (PLHIV), driven by education, prior vaccination, and trust in vaccine efficacy, with regional variations [32]. Compared to the current meta-analysis showing a global student acceptance rate of 58.6%, Ugandan students had lower acceptance, while PLHIV rates were closer to the global average. The factors influencing acceptance varied from local to global contexts.

The acceptance of the Human Papillomavirus (HPV) vaccine among college students provides a relevant comparison to the acceptance rates of the Mpox vaccine, as both target sexually transmitted infections. The research shows that awareness and demographic factors significantly influence students’ willingness to vaccinate against HPV. For instance, awareness varies widely, with studies reporting between 58% and 94% of students having prior knowledge of HPV, and gender differences playing a role, with females generally more aware than males [33,34]. Vaccination uptake also shows great variance, from as low as 4.2% to as high as 82%, depending on the population studied. Factors such as educational background, sexual history, and parental influence are pivotal in shaping these attitudes [34]. Additionally, barriers like vaccine safety concerns, cost, and vaccine hesitancy, especially among males due to a lack of awareness about the vaccine’s relevance, are notable [35].

The findings of this systematic review carry profound implications for public health practice and policy, particularly in enhancing Mpox vaccine uptake among students, a group characterized by moderate acceptance rates. This review highlights the necessity for public health campaigns to tackle vaccine hesitancy effectively by delivering clear and accurate information about the benefits and safety of the vaccine. Employing peer-driven education and advocacy could be particularly impactful within academic settings, where peer influence is significant. Given the observed variability in vaccine acceptance rates across different studies, it is imperative that public health strategies are customized to fit the cultural and geographic contexts of various student populations to ensure relevance and effectiveness.

A major strength of this review lies in its inclusive approach, incorporating a diverse array of studies from multiple global regions. This breadth provides a comprehensive overview of Mpox vaccine acceptance among students and enriches the robustness of the findings. The systematic employment of observational studies and the comprehensive searches across several databases enhance the thoroughness of the review. The use of sensitivity analyses and tools like the Doi plot and the Luis Furuya-Kanamori (LFK) index for assessing potential publication bias also adds layers of credibility to the results. These methodologies help in mitigating the effects of bias and provide a clearer picture of the underlying trends in vaccine acceptance.

However, this review is not without its limitations. The significant heterogeneity observed among the included studies, attributable to variations in study designs, sample sizes, and geographic contexts, poses challenges in drawing definitive conclusions about overall vaccine acceptance trends. This heterogeneity suggests that local factors significantly influence vaccine acceptance rates, necessitating tailored public health interventions. The reliance on self-reported data in many studies could introduce social desirability bias, potentially skewing the data toward more socially acceptable responses. Additionally, the predominance of studies from high-income countries may limit the generalizability of the findings to low- and middle-income countries, where vaccine acceptance dynamics and public health infrastructures differ markedly. The exclusion of non-English studies also introduces a cultural and linguistic bias, potentially overlooking relevant data from non-English-speaking regions. Looking forward, it is crucial that future research addresses these gaps. There is a pressing need for more comprehensive data from low- and middle-income countries to provide a more balanced view of global vaccine acceptance. Longitudinal studies that track changes in vaccine acceptance over time, particularly in response to specific public health campaigns or emerging disease outbreaks, could yield valuable insights into the dynamics of student attitudes toward vaccines. These studies would help in understanding how perceptions evolve and what factors most effectively shift attitudes toward vaccination.

Moreover, qualitative research exploring the deeper motivations, fears, and social influences that govern vaccine decision-making among students could provide deeper insights into the root causes of vaccine hesitancy. This understanding could inform more targeted and effective public health interventions. Additionally, standardizing research methodologies across studies would enhance comparability and reliability of future analyses, allowing for more precise and actionable conclusions. While this review provides significant insights into Mpox vaccine acceptance among students and offers a strong foundation for targeted public health strategies, the highlighted limitations and recommendations for future research underscore the need for ongoing investigation. Addressing these research gaps is essential for developing more effective public health strategies and ensuring broader vaccine coverage, ultimately contributing to better control of infectious diseases in highly populated settings such as universities.

## 5. Conclusions

This systematic review and meta-analysis revealed that Mpox vaccine acceptance among students is moderate but varies significantly across different demographics and regions. These results underline the need for public health campaigns to adapt strategies to local cultures and contexts to improve vaccine uptake, focusing on educational outreach to overcome vaccine hesitancy. Further research is essential to explore the behavioral and socio-economic drivers of vaccine acceptance to develop targeted interventions critical for controlling Mpox and ensuring the effectiveness of future vaccination efforts.

## Figures and Tables

**Table 1:** Basic characteristics of included studies.

Author	Country	Study Design	Male (*n*)	Age (Mean Years)	Total Sample Size	Study Outcomes	Results (Adjusted)	Total Modified NOS Score	Key Findings
Abd elhafeez et al., 2023 [20]	Saudi Arabia	Cross-sectional study	882 (46.0%)	21	1919	Mpox knowledge levels among medical students	Income level, access to educational resources	4	Differences in Mpox knowledge among medical students suggest incorporating epidemiology into curricula to enhance disease control.
Hussein et al., 2024 [21]	Egypt	Cross-sectional study	1445 (52.0%)	22 (median)	2780	Psychological factors affecting vaccination decisions	Socio-demographic characteristics, geographic location	6	Socio-demographic characteristics, geographic location, awareness, and past experiences greatly influence vaccination decisions and societal attitudes toward vaccination.
Kaltman et al., 2006 [22]	USA	Cross-sectional study	0 (0%)	18–32	256	Influences on smallpox vaccine decision-making	Psychological stress, health literacy	5	The decision to vaccinate against smallpox is influenced by psychological stress, vaccine perceptions, and perceived threats. Addressing these can increase vaccination rates.
Kumar et al., 2022 [23]	Pakistan	Cross-sectional study	432 (45.7%)	18–22	946	Mpox knowledge and vaccination willingness	Educational attainment, awareness levels	4	Mpox knowledge among university students is moderate, with considerable gaps. Over half are willing to vaccinate, indicating the need for better education and public awareness.
Lounis et al., 2024 [24]	Algeria	Cross-sectional study	28 (14.3%)	<20 and >30	196	Mpox knowledge and support for vaccination	Educational background, awareness	5	Despite limited Mpox knowledge among Algerian students, their vaccination support mirrors global rates, emphasizing the need to enhance education and counter vaccine hesitancy.
Rostkowska et al., 2021 [25]	Germany	Cross-sectional study	555 (30.5%)	23	1821	Perception of vaccine safety and efficacy	Education level, healthcare exposure	6	European students and junior doctors understand vaccine safety and efficacy, but nearly half remain unvaccinated against the flu, highlighting the need for improved vaccine education.
Wang et al., 2024 [13]	China	Cross-sectional study	1265 (33.0%)	<20 and ≥20	3831	Gaps in Mpox knowledge and attitudes toward vaccination	Social media influence, local vs. international health policies	4	University students have gaps in Mpox knowledge but are positive about vaccination. Enhancing education through social media and using diverse vaccines is recommended.
Yang et al., 2024 [26]	China	Cross-sectional study	3712 (84.7%)	16 and > 26	4380	Willingness to receive Mpox vaccine	Sexual orientation, STD history, educational level	5	Willingness to receive the Mpox vaccine among students in southwest China is influenced by sexual orientation and STD history, suggesting the need for targeted educational efforts.

**Table 2:** Subgroup analysis based on geographic region and quality of the studies.

Category	Subgroup	No. of Studies	Sample Size (N)	Pooled Prevalence (95% CI)	*p*-Value
Geographic region	Saudi Arabia	1	1919	3.96% (3.13; 0.29)	<0.01
	Egypt	1	2780	31.98% (30.25; 33.75)	
	USA	1	256	62.89% (56.66; 68.82)	
	Pakistan	1	946	67.65% (64.57; 70.63)	
	Algeria	1	196	48.47% (41.29; 55.70)	
	Germany	1	1821	98.68% (98.05; 99.15)	
	China	2	8211	64.90% (0.00; 100)	
NOS score	Moderate-quality studies	6	11,528	46.90% (13.96; 82.78)	<0.01
	High-quality studies	2	4601	85.53% (0.00; 100)	

## Data Availability

All data generated or analyzed during this study are included in this published article (and its Supplementary Information Files).

## Supplementary Materials

The following supporting information can be downloaded at: https://www.mdpi.com/article/10.3390/vaccines13020183/s1, Table S1: PRISMA checklist; Table S2: Inclusion and Exclusion criteria; Table S3: The adjusted search terms as per searched electronic databases; Table S4: Modified-Newcastle-Ottawa Scale (NOS).
